# Designing research on concussion treatment in the military setting: Important challenges to consider

**DOI:** 10.1016/j.pmedr.2021.101603

**Published:** 2021-10-18

**Authors:** Rosemay A. Remigio-Baker, Seth Kiser, Hamid Ferdosi, Keith Stuessi, Stephanie Maxfield-Panker, Sidney R. Hinds II

**Affiliations:** a Traumatic Brain Injury Center of Excellence (TBICoE), Silver Spring Metro Center I, 1335 E. W. Hwy, Silver Spring, MD 20910, USA; b General Dynamics Information Technology, 3150 Fairview Park Dr., Falls Church, VA 22042, USA; c Naval Hospital Camp Pendleton, 2016 San Jacinto Rd., Camp Pendleton, CA 92055, USA; d Uniformed Services University, 4301 Jones Bridge Rd., Bethesda, MD 20814, USA

**Keywords:** Military Research, Rehabilitation, Concussion, Research Design, Challenges, IRB, Institutional Review Board, MHS, Military Health System, MTF, Military Treatment Facilities, PCS, Permanent Change of Station, ADSM, Active Duty Service Members, TBI/mTBI, Traumatic Brain Injury / mild Traumatic Brain Injury

## Abstract

Traumatic brain injury (TBI) is highly prevalent on a global scale with concussion being the most common of all TBIs. Concussion research on rehabilitation and symptom resolution is well-established, particularly among athletes, but less is known about the recovery of active duty service members (ADSMs). In a population whose occupational environment increases risk for such injury, it is imperative that treatment is optimized to achieve successful rehabilitation. The military setting, however, has challenges that must be accounted for when developing proposals to study the benefits of new interventions or treatment. This review will provide a discussion on those challenges to better understand the considerations that are essential during the developmental phase of concussion studies within the military setting. This review aims to provide investigators novice to the military setting an overview of considerations when researching concussion among ADSMs.

## Highlights

• Overview of lessons learned from concussion research using military personnel.
• Challenges to conducting concussion research in the military setting.
• Considerations for successfully navigating new research in the military setting.

## 1 Introduction

Traumatic brain injury (TBI) serves as a significant burden worldwide, with an estimated 69 million individuals sustaining a TBI annually. (Dewan et al., 2018) Since 2000, of the over 430,000 cases of TBI that have been diagnosed among active duty service members (ADSMs), 82.4% have been classified as mild in severity (mTBI – also known as concussion). (Traumatic Brain Injury Center of Excellence, 2019) There is growing literature on the impact of mTBI and rehabilitative measures among the general population, particularly athletes. (Broglio et al., 2015, Brown et al., 2014, Leddy et al., 2010, Majerske et al., 2008, Moser et al., 2012) Less is known among military populations (Remigio-Baker et al., 2021, Remigio-Baker et al., 2020a, Remigio-Baker et al., 2019), which may have specific advantages and/or disadvantages regarding the course of recovery and rehabilitation after concussion. Given the demands of military occupation (e.g., unit training/duties, combat, physical training), more research is needed to improve treatment, ensure proper return to duty, and maximize the potential to meet the demands of mission assignments while serving in the military (or force readiness). (Congressional Research Service, 2020)

Conducting research in a military setting requires additional understanding of an environment containing distinct and similar elements of a predominantly civilian population. Such understanding is essential to the potential success of a study’s execution and conduct. Additionally, study findings may impact the subsequent administration/implementation of health care guidelines and, ultimately, impact health outcomes. In this review, we elaborate on recruitment challenges, attrition/compliance, and education to highlight military-specific considerations when conducting research in an active duty military population within the framework of the Military Health System (MHS) delivery of healthcare.

## 2 Recruitment challenges

The validity of a study and its findings depend on the quality of the data collected and the representativeness of the study participants to generalize results across the targeted population.

### 2.1 Chain of command

When recruiting from a military population, awareness of chain of command and perceived/actual power differences are essential in order to avoid sampling bias and undue influence. Junior enlisted members of the military are taught to respect chain of command and adhere to orders given by superiors, whether enlisted or commissioned. Junior enlisted ADSMs may feel compelled by senior active duty providers to participate in a study, or may feel obligated to participate in studies touted by their superiors. This highlights an advantage in having a civilian provider to engage in recruitment. Nonetheless, ethical guidelines (Office of Human Research Protections, 2021) and additional safeguards for ADSMs whose compliance may likely be influenced by the hierarchical environment of the military are mandated by the Institutional Review Boards (IRB). (Office of the Federal Register et al., 2021) Care should be taken in the recruitment process to thoroughly confirm that there is no obligation to participate in any study.

### 2.2 Military Life/Schedules

Active duty study participants must abide by strict, unpredictable military schedules and policies prior to and during a study. Such schedules have significant influence on protocol compliance. Moreover, military mission requirements may supersede the willingness or ability to fully comply in a research study. This is often caused by ADSM training requirements, deployment, or performance of other missions. Therefore, study design/plan should include flexibility where feasible to account for interruptions in protocol completion.

### 2.3 Patient density fluctuations

Based on schedules of deployment, military treatment facilities (MTFs) have periods that fluctuate between high to low density patient flow (i.e., operational tempo). These fluctuations are often unpredictable. Thus, recruitment in military concussion studies have been performed in a “catch-all” manner (Gregory et al., 2017, Remigio-Baker et al., 2020b), in which all opportunities of encounters were targeted and not limited to timing. Unfortunately, this strategy hinders investigators from knowing the number of potential participants lost due to lack of interest or scheduling conflict. Lacking such data prevents differentiation of characteristics between participants and non– participants in studies. Conversely, this strategy accommodates schedule limitations of ADSMs while providing the opportunity to recruit a sufficient sample size to detect potential interventional effects and accommodate attrition. Research findings within the military setting often contribute to changes in clinical practice. In this way, improving the ecological validity of a study will result in outcomes more readily translatable to the conditions of the MTF.

### 2.4 Active duty and civilian providers

It is important to consider how provider experience plays a role in study recruitment. Active-duty providers may have personal experiences with mTBI through military deployment, combat/training, and its management within the military. This may lead to more rigorous recruitment attempts. These individuals may utilize more/other clinical algorithms or resources, push harder for diligent treatment of mTBI study participation, or have the ability to build stronger rapport with command/leadership. Alternatively, civilian providers do not get deployed, allowing for less interrupted participation in studies, particularly important in research that calls for lengthier commitment. The ability to stay in a location for a longer period allows civilian providers a more intimate understanding of the patient ebb and flow, and more time to establish rapport with civilian leadership and staff.

## 3 Attrition AND compliance

Unlike the general population, ADSMs follow strict schedules and assignment change that may significantly deplete sample size towards the end of the study due to attrition, and/or impact study compliance of participants.

### 3.1 Permanent change of station

Attrition due to change in assignment, detail or transfer of an ADSM or military unit to a different duty station (i.e., “permanent change of station,” PCS) is a major challenge when collecting longitudinal data from patients and providers. Additionally, when ADSMs complete contractual service commitments (or End of Active Service), retires or are administratively removed from active duty service, participation in military-related studies decrease. Although this population may experience attrition similar to that of the general population (e.g., lost interest in the study, improved), additional attrition due to the demands of military personnel ‘reshuffling’ make data collection and its maintenance during longitudinal studies very challenging. As is typically done with the general population, a 20% increase in calculated sample size is considered to address issues of loss to follow-up for longitudinal studies. To account for the additional attrition of a military population due to PCS, this percentage may be increased, though no suggested percentages for the military population currently exist. Studies that evaluate such need are warranted. Another strategy to address data attrition is to not restrict the study sample size and gather information on as many individuals that consent to participate. In previous military concussion studies, this was done by a “catch-all” recruitment where individuals were recruited from many military venues and at different time periods. (Gregory et al., 2017, Remigio-Baker et al., 2020b) It is important to note, however, that attrition due to PCS is not voluntary nor systematic, thus mitigating selection bias as attrition is somewhat randomized.

### 3.2 Data Sharing Agreements and Memoranda of Agreements

Theoretically, the military community is a more closed/controlled sample compared to the general population, potentially giving investigators false impression they are at an advantage tracking down subjects lost to follow-up due to military-specific changes in scheduling. However, study approval is granted to a specific location within the MHS. When a participant departs for another catchment area, the approval for that study does not follow the participant. This can be overcome with Data Sharing Agreements and Memoranda of Agreements between MTFs willing to become additional study sites. This is not a simple nor automatic process and may involve additional study review by the IRB overseeing alternate locations. Additionally, as the primary mission of the MHS is healthcare as opposed to research, the support to continue data collection may be limited at additional sites. Verbiage can also be included in the protocol of long-term studies to permit returning ADSMs to re-consent following PCS. This option has been effectively used by studies previously for ADSMs who return from deployment to a MTF approved for data collection. (Remigio-Baker et al., 2020b, Remigio-Baker et al., 2020c) Failure to incorporate such verbiage in the initial IRB-approved protocol would require an amendment, risking additional PCS or the delay of potential treatment and data capture.

### 3.3 Team mentality

Within military culture, participants may attempt to hasten return to duty because of a strong sense of unit inclusion or the belief that they have an obligation to support and assist their “battle buddies” in military training and operations. The desire for ADSMs to return to their military “team” may manifest as non-compliance with study protocols, greater drop-out, or minimizing potential negative effects or ongoing symptoms experienced. Early and ongoing participant education and awareness of the study schedule, what to expect, and the potential benefit for larger knowledge that may result from the study may help mitigate study drop-out.

### 3.4 Compliance of command

ADSM study participation is not only dependent on individual interest, but may be influenced by command (or those who supervise the training/work of ADSMs). Command involvement early in the planning process is imperative, as they will be aware of training schedules, manning and other logistical obstacles that may affect mTBI research. Command “buy-in” should be considered a starting point when developing study proposals. This can be achieved by making command/leadership aware of non-medical and medical policies, and that research may contribute to knowledge that ultimately benefits command mission and combat readiness. Establishing working relationships with commands, particularly for those who have “boots on the ground,” can help the researcher anticipate the fluctuations in patient flow density (e.g., cycles of routine trainings, deployments). Effective communication with supervisors is important prior to the beginning of the study and throughout. Military commands often change, thus, knowledge of the study and its importance must be maintained through such transition. Given successful rapport is established, communication between command and study leads may include reporting of change in command that trigger a process in which rapport can be established with the new supervisor and procedures for education can take place.

Although previous concussion studies in the military may have established strong rapport with site leadership during proposal development, a built-in education component specific for the commands was not usually included, which could have attenuated data attrition. (Gregory et al., 2017, Remigio-Baker et al., 2020b) Such education of command is necessary as it provides a better understanding of the importance of such research work among ADSMs and force readiness. As ADSMs update their supervisor on their mTBI treatment, information on study participation is often also discussed. If “buy-in” is not obtained prior to this discussion, it may make it difficult for ADSMs to continue with study follow-up. Military commitment and priorities might be perceived to be (or actually) undermined, and return to duty/activities may occur prematurely. Military command may not fully understand the importance of concussion research, but nevertheless have a strong influence on ADSM compliance with interventions/treatment.

## 4 Education

Education and its implementation are demonstrated effective means that impact patient compliance and provider practice. (Gold and McClung, 2006, Ockene and Zapka, 2000)

### 4.1 Countering concussion Non-disclosure

Although specific measures required by IRB to secure patient data allow participants some peace of mind in providing sensitive data, the threat of confidentiality breach of sensitive data, such as disclosure of concussion history, which is perceived to be detrimental to obtaining opportunities such as optimized health care or promotions (Foster et al., 2019), may dissuade potential participants to provide such history. In a study of “perceived cost and rewards” for disclosing a concussion, the strongest predictor for disclosure outcome was the belief of negative career outcomes. (Foster et al., 2019) Although there were differences in education provided to concussed patients, having multiple exposure to concussion education, nonetheless, promoted disclosure of concussion. (Register-Mihalik et al., 2020) Although these findings were from the Concussion Assessment, Research and Education (CARE) Consortium Study which is of cadets/officers who do not represent the majority of the military enlisted/non-commissioned officers (i.e., ADSM), fear of negative career outcomes may be a universal reason for non-disclosure of concussion history.

### 4.2 Implementation

Equally significant to the success of a study whose focus is health outcomes is the manner in which educational content is implemented. When education is a part of an intervention being evaluated, having the option of both in-person and virtual asynchronous training may optimize access to a population with varying schedules (such as military personnel) and provide the broadest distribution of trainings. One approach with demonstrated success with military personnel during in-person sessions is the train-the-trainer method in which a subject-matter expert trains additional staff in the delivery of an educational intervention to further widen the distribution of such education. (Remigio-Baker et al., 2020b) As a virtual approach is a relatively novel means to distribute an educational intervention, a comparison between an in-person and virtual delivery has yet to be conducted. It is important to understand differences specific to each approach. Further research is warranted to evaluate differences among in-person training, synchronous virtual training, and asynchronous virtual training to optimize the effectiveness of clinical interventions on mTBI treatment in the military setting.

## 5 Concluding thoughts

Conducting research in the military setting is particularly challenging, especially for investigators unaware of considerations that must be addressed for successful study progression. This report provides a summary of a few considerations for planning mTBI research among ADSMs. Such considerations may help avoid obstacles, prevent study delays and, ultimately, optimize success of research conducted in the military setting.

## Declaration of Competing Interest

The authors declare that they have no known competing financial interests or personal relationships that could have appeared to influence the work reported in this paper.
